# *CD3Z* Genetic Polymorphism in Immune Response to Hepatitis B Vaccination in Two Independent Chinese Populations

**DOI:** 10.1371/journal.pone.0035303

**Published:** 2012-04-18

**Authors:** Li-Ping Pan, Wei Zhang, Li Zhang, Xiao-Pan Wu, Xi-Lin Zhu, Bing-Yu Yan, Jing-Yun Li, Ai-Qiang Xu, Ying Liu, Hui Li

**Affiliations:** 1 National Laboratory of Medical Molecular Biology, Institute of Basic Medical Sciences, Chinese Academy of Medical Sciences, School of Basic Medicine, Peking Union Medical College, Beijing, China; 2 Beijing Center for Disease Control and Prevention, Beijing, China; 3 Shandong Center for Disease Control and Prevention, Jinan, China; 4 Department of Epidemiology, Institute of Basic Medical Sciences, Chinese Academy of Medical Sciences, School of Basic Medicine, Peking Union Medical College, Beijing, China; University of Alabama at Birmingham, United States of America

## Abstract

Vaccination against hepatitis B virus is an effective and routine practice that can prevent infection. However, vaccine-induced immunity to hepatitis B varies among individuals. CD4^+^ T helper cells, which play an important role in both cellular and humoral immunity, are involved in the immune response elicited by vaccination. Polymorphisms in the genes involved in stimulating the activation and proliferation of CD4^+^ T helper cells may influence the immune response to hepatitis B vaccination. In the first stage of the present study, a total of 111 single nucleotide polymorphisms (SNPs) in 17 genes were analyzed, using the iPLEX MassARRAY system, among 214 high responders and 107 low responders to hepatitis B vaccination. Three SNPs (rs12133337 and rs10918706 in *CD3Z*, rs10912564 in *OX40L*) were associated significantly with the immune response to hepatitis B vaccination (*P* = 0.008, 0.041, and 0.019, respectively). The three SNPs were analyzed further with the TaqMan-MGB or TaqMan-BHQ probe-based real-time polymerase chain reaction in another independent population, which included 1090 high responders and 636 low responders. The minor allele ‘C’ of rs12133337 continued to show an association with a lower response to hepatitis B vaccination (*P* = 0.033, odds radio = 1.28, 95% confidence interval = 1.01–1.61). Furthermore, in the stratified analysis for both the first and second populations, the association of the minor allele ‘C’ of rs12133337 with a lower response to hepatitis B vaccination was more prominent after individuals who were overweight or obese (body mass index ≥25 kg/m^2^) were excluded (1^st^ stage: *P* = 0.003, 2^nd^ stage: *P* = 0.002, *P*-combined = 9.47e-5). These findings suggest that the rs12133337 polymorphism in the *CD3Z* gene might affect the immune response to hepatitis B vaccination, and that a lower BMI might increase the contribution of the polymorphism to immunity to hepatitis B vaccination.

## Introduction

Infection with hepatitis B virus (HBV) is a public health problem that seriously threatens human life. It results in more than half a million deaths each year, which are caused mainly by the consequences of the infection, such as chronic hepatitis B, cirrhosis, and hepatocellular carcinoma [1], [2]. Approximately 93 million Chinese people are carriers of HBV, and 300,000 deaths occur every year in China as a result of HBV infection [3]. Recombinant vaccines against HBV have been used since the 1980s and are an effective method of preventing HBV infection and transmission. However, 5–10% of healthy adults fail to produce protective levels of antibody against the hepatitis B vaccine (anti-HBs).

Physical characteristics such as older age, male gender, higher body mass index (BMI), and a history of smoking are associated with a decreased antibody response to hepatitis B vaccination [4], [5], [6], [7]. An increased dose of the hepatitis B vaccine can induce stronger immunity [8]. However, a study of twins indicated that genetic variation accounts for more than 77% of all factors that influence the individual response to hepatitis B vaccination [9]. Several polymorphisms in genes of the major histocompatibility complex (*MHC*) have been reported to be associated with variations in vaccine-induced immune response [10], [11], [12], [13], [14], [15]. Furthermore, polymorphisms in cytokine and cytokine receptor genes, such as the interleukins (*IL*)*1β* [16], *IL4* [17], *IL4R* [18], *IL10* [19], and *IL12B* [20], are associated with different levels of immune response to hepatitis B vaccination. It have been also shown that the interaction between *IL12A* and *IL12B* might influence vaccine-induced immunity against HBV [21].

CD4^+^ T helper (Th) cells play an important role in both cellular immunity, which is mediated by T lymphocytes, and humoral immunity, which is mediated by B lymphocytes [22]. Furthermore, CD4^+^ Th cells are involved in the immune response that is elicited by vaccination [23], [24], [25]. Consequently, the activation and proliferation of CD4^+^ Th cells are important steps in the immune response. The interaction of the T cell receptor (TCR)/CD3 complex on the surface of CD4^+^ Th cells with the MHC-II-peptide complex on the surface of antigen-presenting cells (APCs), which is promoted by CD4, triggers the first signal for the activation of CD4^+^ Th cells [26], [27], [28]. The TCR/CD3 complex comprises the clonotypic TCR α/β subunits, which recognize the MHC-peptide complex, and the invariant subunits γ, δ, ε, and ζ (CD3G, CD3D, CD3E, and CD3Z), which mediate signal transduction [29], [30]. The CD3 complex is indispensable for the activation of CD4^+^ Th cells, because the signal transduction depends on immune receptor tyrosine-based activation motifs (ITAMs) that are found in the CD3 complex [31]. Many changes in the sequences of the genes for TCR and CD3 molecules can affect the expression, structure, assembly or signal transduction of the TCR/CD3 complex, and consequently they have an impact on the activation of CD4^+^ Th cells and then affect the immune response to vaccination [26]. In addition, completion of the activation of CD4^+^ Th cells requires a second signal that is initiated by the interaction between costimulatory molecules on the surface of both APCs and CD4^+^ Th cells [32], [33], [34]. The interaction between CD28/CTLA4 and the ligand CD80/CD86 is a well-known costimulatory signal that initiates the positive/negative stimulation of CD4^+^ Th cell activation [35], [36]. In addition to the classic costimulatory molecules, others such as OX40 and its ligand OX40L, CD54 and its ligand LFA-1 (ITGAL and ITGB2), CD40 and CD40L, and CD58 and CD2 are also important in regulating the activation of CD4^+^ Th cells [37], [38], [39], [40]. It has been reported that single nucleotide polymorphisms (SNPs) in the *ITGAL* gene and haplotypes of the *CD58* and *CD44* genes showed significant associations with anti-HBs levels in a Gambian population [41], [42]. However, there is no available information on the association between polymorphisms in the genes that encode the subunits of the TCR/CD3 complex and costimulatory molecules, and immunity induced by the hepatitis B vaccine in the Chinese Han population.

In the present study, two independent case–control populations were recruited to evaluate whether polymorphisms in the genes that encode the subunits of the TCR/CD3 complex and costimulatory molecules were associated with different outcomes of hepatitis B vaccine-induced immunity in the Chinese Han population. The findings suggested that molecules involved in the activation of CD4^+^ Th cells might influence the efficacy of hepatitis B vaccination, and contributed to a better understanding of the diversity and complexity of the genetic factors that affect the efficacy of hepatitis B vaccination. Furthermore, the results might be helpful in the identification of specific genes for use as targets in the development of novel and more effective vaccines.

## Materials and Methods

### Study populations

The present study was conducted in two independent Chinese Han populations. Participants in the first stage were recruited from among healthy volunteers in the community in Beijing. A total of 1,600 individuals were recruited initially, in 2007, after written informed consent had been obtained. Each participant completed a questionnaire that included questions about demographic information, smoking history, vaccination history, chronic disease, and immunosuppressive disease/medications. All individuals were tested for five markers of hepatitis B using an Abbott i2000 detection kit (Abbott Laboratory, Chicago, IL). Individuals who were negative for the five markers of hepatitis B were tested further for HBV DNA and for anti-HCV and anti-HIV. Participants were excluded if: 1) they were positive for HBV DNA, hepatitis B surface antigen (HBsAg), hepatitis B e antigen (HBeAg), anti-HBs, anti-HBc, anti-HBe, anti-HCV, and/or anti-HIV; 2) they had been vaccinated previously with any hepatitis B vaccine; 3) they had a chronic disease, such as diabetes, cancer, or cardio-cerebrovascular disease, or were undergoing renal dialysis; 4) they had any immunosuppressive disease or were taking any immunosuppressive medication; 5) they were not of Han ethnicity; 6) they were younger than 18 years. The remaining 599 individuals were administered three doses of hepatitis B vaccine at 0, 1, and 6 months (3×10 µg i.m.; North China Pharmaceutical Co., Ltd., Beijing, China). Levels of anti-HBs were measured at 7 months using the Abbott i2000 detection kit. Participants who showed low levels of anti-HBs (10–99 mIU/mL) were designated as low responders, whereas participants who showed high levels of anti-HBs (≥1,000 mIU/mL) were designated as high responders [10]. The definition of low and high responders was based on the level of anti-HBs 1 month after the third dose of vaccine.

Participants in the second (confirmatory) stage of the study were recruited from a large hepatitis B vaccination campaign in Shandong province in 2009. Written informed consent and completed questionnaires were obtained from all participants. The exclusion criteria were the same as those for the Beijing study population. A total of 3985 qualifying individuals remained in the confirmatory population. They were divided randomly into three subgroups and administered different recombinant hepatitis B vaccines (1st group: 3×20 µg yeast-derived recombinant hepatitis B vaccine, GlaxoSmithKline Investment Co., Ltd., UK; 2nd group: 3×20 µg CHO-derived recombinant hepatitis B vaccine, North China Pharmaceutical Co., Ltd., Beijing; 3rd group: 3×10 µg yeast-derived recombinant hepatitis B vaccine, Dalian Hissen Bio-pharmaceutical Co., Ltd., Dalian.). The vaccination schedule, the method of detection of anti-HBs, and the definitions of low and high responders were the same as for the Beijing study population described above. All low responders were included in this study, while high responders were selected randomly until a similar age and gender ratio was attained for the high responder group as was present in the low responder group. The process of sample selection were: high and low responders were categorized into three age groups (19–30 years old, 31–40 years old, and 41–50 years old), respectively, and then the high responders in each age group were selected randomly to reach a similar gender ratio to that of the corresponding low responder age group. The statistical software SPSS (version 11.0) was used to evaluate the characteristics of the sample.

The study was performed in accordance with the guidelines of the Helsinki Declaration and was approved by the Ethics Committee of the Institute of Basic Medical Sciences, Chinese Academy of Medical Science.

### Selection of candidate genes and SNPs

In the first stage, a total of 111 SNPs in 17 genes were genotyped. These genes were selected on the basis of their function in regulating the activation and proliferation of CD4^+^ Th cells. The proteins encoded by the *CD3G*, *CD3D*, *CD3E*, *CD3Z*, and *CD4* genes are indispensable co-receptors that help T cell receptors to combine with the MHC-peptide complex and mediate intracellular signal transduction, which is considered to be the first signal for activation of CD4^+^ T cells. Costimulatory molecules were selected on the basis of their function in initiating the essential second signal that stimulates the activation of CD4^+^ T cells. The Tagger Pairwise Tagging protocol in HapMap was used to select tag SNPs within each gene, using the following conditions: Chinese Han race in Beijing, China (CHB), r^2^>0.8, and a minor allele frequency (MAF) >0.05 (HapMap Data Rel 24/Phase II, November 2008, on NCBI B36 assembly, dbSNP b126). Other SNPs within each gene were chosen on the basis of their probable functional importance and a MAF >0.05. A full list of the 111 SNPs is given in Table S1. The SNP ID numbers and detailed sequence information are available at http://www.ncbi.nlm.nih.gov/SNP/. The SNPs that showed significant *P* values (<0.05) in the first stage were further analyzed in the second stage.

### DNA amplification and SNP genotyping

Genomic DNA was extracted from peripheral blood using the phenol–chloroform method. In the first stage, the 111 SNPs were genotyped using the iPLEX MassARRAY system (Sequenom Inc., San Diego, CA, USA). The details of the primer sequences that were used for genotyping are summarized in Table S1. To minimize bias in genotyping errors during the first stage, a mix of samples from high responders and low responders were included on each plate. The SNPs with *P* values<0.05 were selected for follow-up analysis during the second stage using the TaqMan-MGB (Genecore Biotech Co., Ltd., Shanghai, China) or TaqMan-BHQ (Sangon Biotech Co., Ltd., Shanghai, China) probe-based real-time polymerase chain reaction (PCR). The primer and probe sequences that were used to genotype each SNP during the second stage are shown in Table S2. Amplification and detection were conducted using a Bio-Rad iQ5 Multicolor Real-Time PCR Detection system (Bio-Rad, Hercules, CA). To confirm the genotyping results from the first stage, 5% of the samples from Beijing population, selected randomly, were replicated with the TaqMan-MGB or TaqMan-BHQ probes that were used in the second stage.

### Statistical analysis

A χ2 goodness-of-fit test was used to examine whether the genotype distributions of each SNP conformed to Hardy–Weinberg equilibrium (HWE) in the whole study population. The EPI software (version 6.0) was used to calculate the statistical power. The allele frequencies for each SNP were compared between the high and low responders using the χ2 test. The genotype distributions of each SNP were calculated using the Cochran–Armitage trend test; the calculation was conducted with EPI software (version 6.0). Logistic regression analysis was used to adjust for confounding factors, such as age, gender, BMI, smoking history, and vaccines; this analysis was performed using SPSS software (version 11.0). Linkage disequilibrium of the *CD3Z* gene was tested using Haploview (version 4.2). The threshold for statistical significance was *P*<0.05.

## Results

### Demographic characteristics of the two study populations

Among the 599 participants who were analyzed in the first stage, 107 individuals showed low levels of anti-HBs (10–99 mIU/mL) and were assigned to the low responder group, whereas 214 individuals showed high levels of anti-HBs (≥1,000 mIU/mL) and were assigned to the high responder group [10]. Among the 3985 individuals in the second study population, 636 individuals were low responders (anti-HBs: 10–99 mIU/mL), and 1090 individuals were selected from among the high responders (anti-HBs ≥1,000 mIU/mL) to give a control group that had similar age and gender ratios to those of the low responder group.

The demographic details of the two study populations are summarized in Table 1. All of the participants belonged to the Chinese Han population. In the first study population, age, gender, BMI, and smoking status differed significantly between the high and low responders (*P*<0.05). There were no significant differences in the demographic characteristics of high and low responders in the second study population (*P*>0.05).

**Table 1:** Characteristics of the study populations.

	Beijing			Shandong		
	HR n = 214 (100%)^b^	LR n = 107 (100%)^c^	*P*	HR n = 1090 (100%)^b^	LR n = 636 (100%)^c^	*P*
**Age (years)** a	35.2±7.70	38.2±6.71	<0.001	40.1±7.08	40.6±6.93	0.12
**Gender**						
Male	81 (37.9)	59 (55.1)	0.003	553 (50.7)	337 (53.0)	0.37
Female	133 (62.1)	48 (44.9)		537 (49.3)	299 (47.0)	
**BMI (kg/m^2^)** a	23.3±3.44	24.7±3.57	0.001	24.4±3.05	24.6±3.09	0.08
**<25**	151 (70.6)	55 (51.4)	<0.001	702 (64.4)	382 (60.1)	0.07
**≥25**	63 (29.4)	52 (48.6)		388 (35.6)	254 (39.9)	
**Smoking**						
Yes	37 (17.3)	44 (41.1)	<0.001	183 (16.8)	118 (18.6)	0.35
No	177 (82.7)	63 (58.9)		907 (83.2)	518 (81.4)	
**Vaccines** d						
**1**	-	-		408 (37.4)	236 (37.1)	
**2**	-	-		368 (33.8)	188 (29.6)	0.09
**3**	-	-		314 (28.8)	212 (33.3)	
**4**	214 (100)	107 (100)	-			

a Values are mean ± SD.

b HR: High responder (anti-HBs ≥1000 mIU/mL).

c LR: Low responder (anti-HBs 10–99 mIU/mL).

d Vaccines: 1) Recombinant yeast-derived hepatitis B vaccine, 3×20 µg. 2) Recombinant CHO-derived hepatitis B vaccine, 3×20 µg. 3) Recombinant yeast-derived hepatitis B vaccine, 3×10 µg. 4) Recombinant CHO-derived hepatitis B vaccine, 3×10 µg.

### Association between SNPs and the immune response to hepatitis B vaccination

#### Analysis of the 1^st^ stage

A total of 111 SNPs were genotyped during the first stage, but four SNPs were not genotyped successfully and were excluded from the analysis. Of the remaining 107 SNPs, the average success rate for genotyping was 99.1% (ranging from 71.3–100%). Two SNPs had call rates below 90% and were excluded from the analysis. Hence, 105 SNPs were analyzed in the first stage (Table S3). Conformity to HWE was analyzed for the whole study population. Of the 105 SNPs, 15 SNPs did not conform to HWE (*P*<0.05). Among the 321 samples analyzed, 318 samples (99%) had a call rate >90%, and 144 samples (45%) had a call rate of 100%.

The genotype distributions of the 105 SNPs were compared between the high responders and low responders and the results are summarized in Table S3. Four SNPs (rs12133337 and rs10918706 in the *CD3Z* gene, rs10912564 in the *OX40L* gene, and rs2298209 in the *OX40* gene) showed significant associations with the immune response to hepatitis B vaccination (Table 2). The SNP rs2298209 is located in the 3′-untranslated region (3′-UTR) of *OX40*, whereas the other three SNPs are tag SNPs located in intron. The frequency of the minor allele ‘C’ of rs12133337 in the *CD3Z* gene was significantly higher in the low responders than in the high responders [14.0% *vs*. 7.5%, *P* = 0.008, odds ratio (OR) = 2.02], and the distribution of the genotypes differed significantly between high responders and low responders (*P* = 0.009). The frequency of the minor allele ‘T’ of the other SNP in the *CD3Z* gene (rs10918706) was significantly higher in the high responders than in the low responders (*P* = 0.041, OR = 0.68). The frequency of minor allele ‘T’ of rs10912564 in the *OX40L* gene was significantly higher in the low responders than in the high responders (*P* = 0.019, OR = 2.60). In addition, the frequency of minor allele ‘C’ of rs2298209 in the *OX40* gene was significantly higher in the low responders than in the high responders (*P* = 0.031, OR = 1.99). Given that the frequencies of occurrence of the risk factors described herein ranged from 0.9% to 49.3%, the present study demonstrated a power to detect an allelic association of 13.4% to 81.2% with an OR of 2.0 at a significance level of 0.05.

**Table 2:** Genotype distributions and allelic frequencies of SNPs associated with response to hepatitis B vaccination in Beijing and Shandong populations.

Results of 1^st^ stage (Beijing population)										
Gene/SNP		Alleles					Genotypes			
		HR^a^	LR^b^	*P*	OR (95%CI)		HR^a^	LR^b^	*P* c	OR (95%CI)^c^
*CD3Z*/rs12133337		n = 214 (100%)	n = 107 (100%)				n = 214 (100%)	n = 107 (100%)		
	T	396 (92.5)	184 (86.0)	**0.008**	2.02	TT	183 (85.5)	80 (74.8)		
	C	32 (7.5)	30 (14.0)		(1.15–3.53)	CT	30 (14.0)	24 (22.4)	**0.016**	1.98
						CC	1 (0.5)	3 (2.8)		(1.13–3.46)
*CD3Z*/rs10918706		n = 213 (100%)	n = 107 (100%)				n = 213 (100%)	n = 107 (100%)		
	C	287 (67.4)	161 (75.2)	**0.041**	0.68	CC	97 (45.1)	61 (57.1)		
	T	139 (32.6)	53 (24.8)		(0.46–1.00)	TC	93 (44.7)	39 (35.9)	**0.020**	0.62
						TT	23 (10.2)	7 (7.1)		(0.42–0.93)
*OX40L*/rs10912564		n = 196 (100%)	n = 93 (100%)				n = 196 (100%)	n = 93 (100%)		
	C	381 (97.2)	173 (93.0)	**0.019**	2.60	CC	185 (94.4)	81 (87.1)		
	T	11 (2.8)	13 (7.0)		(1.07–6.37)	TC	11 (5.6)	11 (11.8)	**0.009**	3.36
						TT	0 (0)	1 (1.1)		(1.36–8.32)
*OX40/*rs2298209		n = 213 (100%)	n = 107 (100%)				n = 213 (100%)	n = 107 (100%)		
	G	405 (95.1)	194 (90.7)	**0.031**	1.99	GG	194 (91.1)	87 (81.3)		
	C	21 (4.9)	20 (9.3)		(1.01–3.92)	CG	17 (8.0)	20 (18.7)	0.152	1.64
						CC	2 (0.9)	0 (0)		(0.83–3.24)

Results of 2^nd^ stage (confirmation in Shandong population)										
Gene/SNP		Alleles					Genotypes			
		HR n = 1090 (100%)^a^	LR n = 636 (100%)^b^	*P*	OR (95%CI)		HR n = 1090 (100%)^a^	LR n = 636 (100%)^b^	*P* d	OR (95%CI)^d^
*CD3Z*/rs12133337	T	1976 (90.6)	1124 (88.4)	**0.033**	1.28	TT	898 (82.4)	498 (78.3)		
	C	204 (9.4)	148 (11.6)		(1.01–1.61)	CT	180 (16.5)	128 (20.1)	**0.037**	1.27
						CC	12 (1.1)	10 (1.6)		(1.01–1.58)

Combined results										
Gene/SNP		Alleles					Genotypes			
		HR n = 1304 (100%)^a^	LR n = 743 (100%)^b^	*P*	OR (95%CI)		HR n = 1304 (100%)^a^	LR n = 743 (100%)^b^	*P* d	OR (95%CI)^d^
*CD3Z*/rs12133337	T	2372 (91.0)	1308 (88.0)	**0.003**	1.37	TT	1081 (82.9)	578 (77.8)		
	C	236 (9.0)	178 (12.0)		(1.11–1.69)	CT	210 (16.1)	152 (20.5)	**0.008**	1.33
						CC	13 (1.0)	13 (1.7)		(1.08–1.63)

a Values are mean ± SD.

b HR: High responder (anti-HBs ≥1000 mIU/mL).

c LR: Low responder (anti-HBs 10–99 mIU/mL).

d Vaccines: 1) Recombinant yeast-derived hepatitis B vaccine, 3×20 µg. 2) Recombinant CHO-derived hepatitis B vaccine, 3×20 µg. 3) Recombinant yeast-derived hepatitis B vaccine, 3×10 µg. 4) Recombinant CHO-derived hepatitis B vaccine, 3×10 µg.

Logistic regression analysis with an additive model was used to adjust for the confounding factors. As shown in Table 2, after adjustment for confounding factors such as age, gender, BMI, and smoking, three SNPs (rs12133337 and rs10918706 in *CD3Z*, rs10912564 in *OX40L*) remained association with the immune response to hepatitis B vaccination (*P* = 0.016, OR = 1.98; *P* = 0.020, OR = 0.62; *P* = 0.009, OR = 3.36, respectively), whereas the SNP rs2298209 did not show a significant association with this response.

#### Analysis of the 2^nd^ stage

The three SNPs that were shown to be associated with the response to vaccination in the first stage of the study (rs12133337 and rs10918706 in *CD3Z*, rs10912564 in *OX40L*) were genotyped in the second stage. The call rate for each SNP was 100%. A total of 1090 high responders and 636 low responders were included in the second stage. The genotype distributions of all three SNPs did not deviate from HWE (*P*>0.05). A summary of the results of the single SNP analysis in the second stage is shown in Table 2 and Table S4. Among the three SNPs, we detected one SNP in the *CD3Z* gene (rs12133337) that showed an association with the immune response to hepatitis B vaccination. The frequency of the minor allele ‘C’ of rs12133337 was significantly higher in the low responders than in the high responders (11.6% *vs*. 9.4%, *P* = 0.033, OR = 1.28), and the distribution of genotypes differed significantly between high responders and low responders (*P* = 0.035). Logistic regression analysis with the additive model was used to adjust for confounding factors. As shown in Table 2, after adjustment for confounding factors such as age, gender, BMI, smoking, and vaccines, the minor allele ‘C’ of rs12133337 retained its association with a lower immune response to hepatitis B vaccination (*P* = 0.037, OR = 1.27). These findings were consistent with the results obtained in the first stage. Next, the two populations were combined in the analysis, and we observed that the frequency of the minor allele ‘C’ of rs12133337 was also significantly higher in the low responders than in the high responders in the combined sample (12.0% *vs*. 9.0%, *P* = 0.0028, OR = 1.37).

Further stratified analysis showed that in both the first and the second stage, the effect of rs12133337 was more evident in participants who had a lower BMI (<25 kg/m^2^), which implicated that lower BMI might increase the contribution of rs12133337 to the immune response to hepatitis B vaccination. As shown in Table 3, after adjustment for confounding factors by the use of logistic regression with a dominant model, the frequency of the minor allele ‘C’ of rs12133337 was significantly higher in the low responders than in the high responders in Beijing population (*P* = 0.003, OR = 3.58). Similar results were observed in Shandong population (*P* = 0.002, OR = 1.65). After the two populations had been combined, we found that the frequency of the minor allele ‘C’ of rs12133337 was also significantly higher in the low responders than in the high responders (*P*-combined = 9.47e-5, OR = 1.81).

**Table 3:** Stratified analysis of rs12133337 effects on the immune response to hepatitis B vaccination by BMI.

	BMI ≥25 kg/m^2^				BMI <25 kg/m^2^			
1^st^ stage								
	HR n = 63 (100%)^a^	LR n = 52 (100%)^b^	*P* c	OR (95%CI)^c^	HR n = 151 (100%)^a^	LR n = 55 (100%)^b^	*P* c	OR (95%CI)^c^
TT	48 (76.2)	40 (76.9)	0.98	1.01	135 (89.4)	40 (72.7)	**0.003**	3.58
CT+CC	15 (23.8)	12 (23.1)		(0.98–1.12)	16 (10.6)	15 (27.3)		(1.53–8.38)

2^nd^ stage								
	HR n = 388 (100%)^a^	LR n = 254 (100%)^b^	*P* d	OR (95%CI)^d^	HR n = 702 (100%)^a^	LR n = 382 (100%)^b^	*P* d	OR (95%CI)^d^
TT	302 (77.8)	203 (79.9)	0.43	0.85	596 (84.9)	295 (77.2)	**0.002**	1.65
CT+CC	86 (22.2)	51 (20.1)		(0.58–1.26)	106 (15.1)	87 (22.8)		(1.20–2.27)

combined								
	HR n = 451 (100%)^a^	LR n = 306 (100%)^b^	*P* d	OR (95%CI)^d^	HR n = 853 (100%)^a^	LR n = 437 (100%)^b^	*P* d	OR (95%CI)^d^
TT	350 (77.6)	243 (79.4)	0.42	0.86	731 (85.7)	335 (76.7)	**<0.001**	1.81
CT+CC	101 (22.4)	63 (20.6)		(0.60–1.23)	122(14.3)	102 (23.3)		(1.34–2.43)

a HR: High responder (anti-HBs ≥1,000 mIU/ml);

b LR: Low responder (anti-HBs 10–99 mIU/ml).

c *P-*values and OR (95% CI) were adjusted for age, gender, and smoking history.

d *P-*values and OR (95% CI) were adjusted for age, gender, smoking history, and vaccines.

Given that rs12133337 is in a non-coding region, linkage disequilibrium of the *CD3Z* gene was tested using the SNPs genotyped during the first stage, to assess the functional relevance of rs12133337. However, no SNP was in linkage disequilibrium with rs12133337 (Figure S1).

## Discussion

Vaccine-induced immunity to hepatitis B is a complex process that is controlled by numerous factors. In addition to environmental factors and host-related physical factors, genetic variation also plays an important role in regulating the magnitude of the immune response to hepatitis B vaccination [9], [12]. As described above, *MHC* loci have attracted a great deal of attention and many consistent results for the role of this region in the immune response to hepatitis B vaccination have been obtained. Furthermore, polymorphisms in families of cytokine genes have been implicated in influencing the degree of immune response to hepatitis B vaccination. However, reports on polymorphisms in other candidate genes, such as T cell co-receptor and costimulatory molecule genes that are involved in regulating the activation and proliferation of CD4^+^ Th cells, are limited [41], [42].

In the present study, we evaluated two independent case–control study populations, and found that the SNP rs12133337 in the *CD3Z* gene was associated with the immune response to hepatitis B vaccination in both populations. The SNP rs12133337 is a tag SNP and is located in the first intron of the *CD3Z* gene on chromosome 1q22–23. Two known SNPs (rs16859085 and rs10918698) that are tagged by rs12133337 are also located in the intron region, and SNPs in the *CD3Z* gene that were genotyped in the present study are not in linkage disequilibrium with rs12133337, which bring out difficulties in functional analysis. However, it cannot be ruled out that other SNPs might be in linkage disequilibrium with rs12133337. CD3Z is an important subunit of the CD3 complex and acts as an amplification module. Three ITAMs in CD3Z are essential for signal transduction, whereas the other subunits of the CD3 complex contain only one ITAM [43]. Phosphorylation of CD3Z at tyrosine residues in the ITAMs occurs at an early stage after TCR engagement, and this is important for signal transduction [44]. The abnormal expression or false assembly of the TCR-CD3 complex can result in T lymphocyte dysfunction [45]. Experiments with CD3Z-deficient animals have shown that the stable assembly and expression of TCR depends on the amount of CD3Z molecules present [46]. It has been reported that an SNP in the intron of the *CD3Z* gene shows an association with rheumatoid arthritis [47], and another SNP in the intron of the *CD3Z* gene is associated with systemic sclerosis [48]. These findings suggest that the CD3Z molecule plays an important role in immunity. Furthermore, decreased expression of *CD3Z* has been detected in patients infected with HCV [49] and HIV [50], which implies that the CD3Z molecule might correlate with progression of infectious disease and that lower expression of *CD3Z* might result in an impaired immune response. However, the detailed mechanism of action of the CD3Z molecule in vaccine-induced immunity is not clear. The SNP rs12133337 is a genetic marker, and we hypothesize that it might be in linkage disequilibrium with other SNPs that affect the expression of *CD3Z* directly, thus these SNPs might regulate the expression of *CD3Z* collectively. Additional study is necessary to clarify the function of CD3Z in the immune response that is elicited by hepatitis B vaccination.

A higher BMI might decrease the immune response triggered by hepatitis B vaccination, and individuals who are overweight or obese are not easy to produce protective anti-HBs after hepatitis B vaccination [4], [5]. The world health organization (WHO) has proposed that a BMI ≥25 kg/m^2^ should be defined as overweight and that ≥30 kg/m^2^ represents obesity [51], [52]. In the present study, we found that in both the Beijing and Shandong populations, the association of the minor allele ‘C’ of rs12133337 with a lower response to hepatitis B vaccination was more prominent after overweight and obese participants had been excluded. This is consistent with the idea that a lower BMI might increase the contribution of the SNP to the immune response to hepatitis B vaccination. However, the strength of the association identified in the stratified analysis differed between participants from Beijing and Shandong (OR = 3.58 vs. 1.65). We compared the proportion of low responders among participants from Beijing and Shandong (17.8% among Beijing participants and 16.0% among Shandong participants) and found no significant difference. Therefore, the difference in the strength of association might be a result of the different sample size, the different demographics, and the different geographic sources between Beijing and Shandong populations. Furthermore, we cannot rule out the possibility that there was a selection bias in our study.

The two populations in the present study showed the effects of rs12133337 on hepatitis B vaccine-induced immunity. Logistic regression analysis was performed to adjust for confounding factors that might have biased the association of the SNP with the immune response to hepatitis B vaccination. Furthermore, the influence of chronic disease was controlled by excluding individuals with chronic diseases from the study. Until now, there have been a limited number of studies of the associations between genetic polymorphisms and the immune response to hepatitis B vaccination in the Chinese Han population [18], [21], and the sample sizes were not sufficiently large. In the present study, two populations, one from Beijing (1^st^ stage) and a second from Shandong (2^nd^ stage), were included, and the size of the Shandong population was sufficiently large to confirm the results obtained during the first stage.

However, several limitations of our study should be addressed. The Shandong population was not a strict replicate population because the demographics, geography, and vaccine regime differed from those of the Beijing population. Only low and high responders were included in our association study [10]. A lack of power caused by the moderate sample size in the first stage prevented us from assessing genetic effects in non-responders to hepatitis B vaccination (anti-HBs <10 mIU/mL). Low responders (anti-HBs 10–99 mIU/mL) are also at risk of infection with HBV owing to waning antibody levels, although the minimum level for clinical protection is accepted to be 10 mIU/mL. It should be considered that the diversity of individual immune responses and subsequent decreases in the levels of anti-HBs, as well as possible errors in the quantitative determination of anti-HBs, might influence individuals that are actually at risk of infection [53], [54]. Furthermore, it has been reported that some countries have adopted a higher reference level for anti-HBs (e.g., 100 mIU/mL in the UK) [55]. Association study that focused on the low response phenotype might contribute indirectly to the prevention of HBV infection in low responders. Given that our study was focused on polymorphisms in the genes that encode the subunits of the TCR/CD3 complex and costimulatory molecules, the SNPs that have been reported previously within the HLA region and other genes associated with hepatitis B vaccine-induced immunity in the Chinese Han population were not included. We cannot rule out the possibility that these SNPs might also affect the immune response to hepatitis B vaccination. The different doses and sources of vaccine that were used in the second study population might have contributed to different levels of efficacy of vaccination, and thus might have biased the outcome of the immune response to hepatitis B vaccine. However, there was no statistically significant difference in the frequencies of high responders and low responders in each vaccine group, and logistic regression analysis was used to adjust for confounding factors and thus helped to eliminate bias. Owing to the modest size of the Beijing population, the statistical power in the first stage would have been decreased for the SNPs with lower MAFs. Thus, the other genes that were tested in the first study population might also affect the immune response to hepatitis B vaccination, even though they did not reach the level of significance. Close family relatives (parent–offspring pairs and siblings) were excluded from our study. However, the second-degree or higher relatives might be present and had an impact on our results. The association identified in the present study should be validated in other ethnic populations. The immune response elicited by vaccination is the cumulative effect of multiple genes in the immune response network [56]. Therefore, in future studies, greater attention should be paid to the combined effects of SNPs in different genes on the efficacy of the immune response to vaccination.

In conclusion, the results of the present study have shown that the minor allele ‘C’ of rs12133337 in the *CD3Z* gene is associated with susceptibility to a lower immune response to hepatitis B vaccination in the Chinese Han population, and that a lower BMI might increase the contribution of the polymorphism to this immune response. These findings suggest that polymorphisms in the *CD3Z* gene might affect the outcome of the immune response to vaccination against hepatitis B. Furthermore, the study suggests that several novel candidate genes outside the HLA region might influence the immunity that is induced by vaccination against hepatitis B. Additional studies that concentrate on the search for new molecules involved in the immune response to hepatitis B vaccination might lead to a better understanding of the mechanism of action of vaccines.

## Supporting Information

Figure S1 **Linkage disequilibrium map of** ***CD3Z*** **gene. Note: Linkage disequilibrium was tested using the SNPs in the** ***CD3Z*** **gene that were genotyped in the first stage. r2 values are shown in the lozenges.**
(TIF)

Table S1 **The details of candidate SNPs and the primer sequences for genotyping in first stage.**
(DOC)

Table S2 **The details of primer and probe sequences for SNP genotyping in the second stage.**
(DOC)

Table S3 The genotype distributions of successfully genotyped SNPs. ^a^ anti-HBs ≥1000 mIU/ml; ^b^ anti-HBs 10–99 mIU/ml. ^*^ deviated from Hardy-Weinberg equilibrium.
(DOC)

Table S4 **Confirmatory study in Shandong population. ^a^ HR: High-response group (anti-HBs ≥1000 mIU/ml). ^b^ LR: Low-response group (anti-HBs 10–99 mIU/ml). ^c^** ***P*** **-values for Cochran-Armitage Trend test.**
(DOC)
