# Supplementary figures and images for: CD3Z Genetic Polymorphism in Immune Response to Hepatitis B Vaccination in Two Independent Chinese Populations
Source: PLoS One. 2012 Apr 18;7(4):e35303. doi: 10.1371/journal.pone.0035303 (PMC3329423; doi:10.1371/journal.pone.0035303)

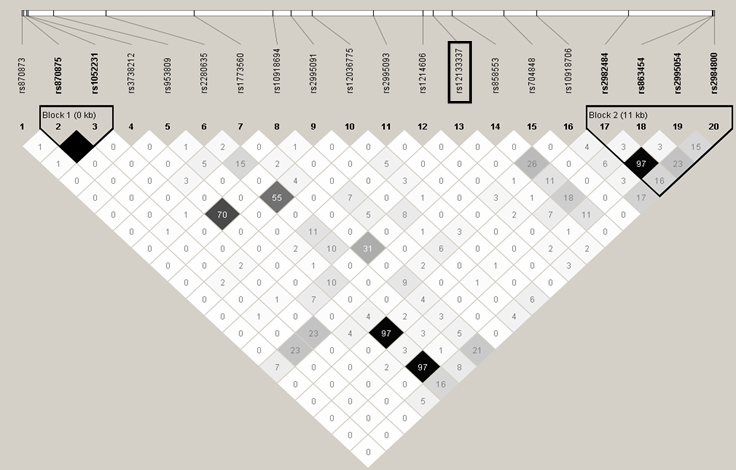

Supplement: Figure S1 — Linkage disequilibrium map of CD3Z gene. Note: Linkage disequilibrium was tested using the SNPs in the CD3Z gene that were genotyped in the first stage. r2 values are shown in the lozenges. (TIF) [file pone.0035303.s001.tif]
