# Supplementary material for: CD3Z Genetic Polymorphism in Immune Response to Hepatitis B Vaccination in Two Independent Chinese Populations
Source: PLoS One. 2012 Apr 18;7(4):e35303. doi: 10.1371/journal.pone.0035303 (PMC3329423; doi:10.1371/journal.pone.0035303)
Supplement: Table S2 — The details of primer and probe sequences for SNP genotyping in the second stage. (DOC) [file pone.0035303.s003.doc]

**Supplementary Table S2** The details of primer and probe sequences for SNP genotyping in the second stage

| **SNPs ID** | **Primer (5'-3')** | **Probe (5'-3')** |
| --- | --- | --- |
| rs12133337 | F:CAGGGTGGTGGTATCTAGACCC | P1:FAM-TTCCCACCAACCTTGAAGA**C**TCTGATATTT-BHQ |
|  | R: TTCCAGCTCCCTTGTGGG | P2:HEX-TTCCCACCAACCTTGAAGA**T**TCTGATATTT-BHQ |
| rs10918706 | F: GGGGTGTGGGTGACGTGTAC | P1:FAM-CCCTGGAA**C**TTAGGAA-MGB |
|  | R: CCCGCCTCCCCGTAAA | P2:HEX-CCCTGGAA**T**TTAGGAAT-MGB |
| rs10912564 | F:ATTGATACACAGCTTTGTAAATGCTAAGAT | P1:FAM-ATGCTTCC**G**TTAACATA-MGB |
|  | R:TGCACACACATACTATAAAGTCAACATTTT | P2:HEX-CATGCTTCC**A**TTAACATA-MGB |
