# Supplementary material for: CD3Z Genetic Polymorphism in Immune Response to Hepatitis B Vaccination in Two Independent Chinese Populations
Source: PLoS One. 2012 Apr 18;7(4):e35303. doi: 10.1371/journal.pone.0035303 (PMC3329423; doi:10.1371/journal.pone.0035303)
Supplement: Table S3 — The genotype distributions of successfully genotyped SNPs. a anti-HBs ≥1000 mIU/ml; b anti-HBs 10–99 mIU/ml. * deviated from Hardy-Weinberg equilibrium. (DOC) [file pone.0035303.s004.doc]

**Supplementary table S3** The genotype distributions of successfully genotyped SNPs

| **Candidate**  **gene** | **db SNP(105)** | **Genotype** | **No. in high-responders ^a^** | **No. in**  **low-responders ^b^** |
| --- | --- | --- | --- | --- |
| ***CD4*** | rs11064391^*^ | AA/AC/CC | 42/54/104 | 12/28/52 |
|  | rs11064392 | GG/GA/AA | 31/94/88 | 14/54/39 |
|  | rs7956804 | GG/GA/AA | 23/86/105 | 12/43/52 |
|  | rs2855534^*^ | CC/CG/GG | 34/72/108 | 13/39/55 |
|  | rs1075837^*^ | CC/CA/AA | 33/85/96 | 16/39/52 |
|  | rs11064410 | GG/GA/AA | 3/64/147 | 2/29/76 |
|  | rs2707212^*^ | AA/AG/GG | 33/79/101 | 14/40/53 |
|  | rs1075835 | TT/TC/CC | 17/88/108 | 13/41/53 |
|  | rs3782736 | CC/CA/AA | 15/100/97 | 8/48/51 |
|  | rs12812942 | TT/TA/AA | 14/85/115 | 4/39/64 |
|  | rs10774451 | AA/AG/GG | 38/105/67 | 17/49/41 |
|  | rs1894290 | GG/GA/AA | 29/87/97 | 11/42/54 |
|  | rs3213427 | CC/CT/TT | 1/31/182 | 0/10/97 |
|  | rs3829972 | AA/AG/GG | 44/105/65 | 19/60/28 |
|  | rs1045261 | CC/CT/TT | 37/111/66 | 19/63/25 |
| ***CD3G*** | rs2239695 | AA/AG/GG | 40/92/82 | 24/48/35 |
|  | rs2071381^*^ | AA/AG/GG | 57/95/61 | 34/46/27 |
|  | rs7947185^*^ | CC/CT/TT | 9/56/148 | 7/26/74 |
|  | rs4544037 | CC/CT/TT | 47/101/66 | 24/53/30 |
|  | rs1561966 | CC/CT/TT | 50/102/61 | 24/48/35 |
| ***CD3E*** | rs3825051 | GG/GA/AA | 57/91/63 | 29/54/24 |
|  | rs2231439 | GG/GA/AA | 20/81/113 | 9/42/56 |
|  | rs2231440 | AA/AG/GG | 14/71/128 | 4/32/71 |
|  | rs1945764 | CC/CT/TT | 18/79/115 | 10/38/59 |
|  | rs3782042 | AA/AG/GG | 54/100/60 | 32/53/22 |
| ***CD3D*** | rs2276424 | TT/TG/GG | 18/88/107 | 4/42/61 |
| ***CD3Z*** | rs863454 | TT/TG/GG | 4/23/187 | 0/16/91 |
|  | rs2995054 | AA/AG/GG | 2/56/156 | 0/19/88 |
|  | rs2984800 | TT/TC/CC | 2/56/156 | 0/19/88 |
|  | rs2995091 | CC/CT/TT | 2/40/171 | 0/22/85 |
|  | rs953809 | TT/TC/CC | 4/68/142 | 6/17/84 |
|  | rs1214606 | AA/AC/CC | 55/100/58 | 24/55/28 |
|  | rs2280635 | GG/GA/AA | 12/63/139 | 7/35/65 |
|  | rs858553 | AA/AG/GG | 6/45/163 | 2/27/78 |
|  | rs1773560 | GG/GA/AA | 7/43/163 | 0/28/79 |
|  | rs2995093 | GG/GA/AA | 34/99/81 | 11/52/44 |
|  | rs10918706 | TT/TC/CC | 23/93/97 | 7/39/61 |
|  | rs704848 | GG/GC/CC | 33/86/94 | 11/53/43 |
|  | rs12133337 | CC/CT/TT | 1/30/183 | 3/24/80 |
|  | rs10918694 | TT/TC/CC | 33/96/84 | 23/45/39 |
|  | rs2982484 | AA/AG/GG | 30/103/81 | 8/53/46 |
|  | rs12036775 | TT/TC/CC | 21/82/110 | 10/46/51 |
|  | rs3738212 | GG/GC/CC | 4/62/148 | 0/34/73 |
|  | rs1052231 | AA/AT/TT | 4/69/141 | 6/18/83 |
|  | rs870875 | CC/CA/AA | 49/99/66 | 22/48/37 |
|  | rs870873 | CC/CT/TT | 5/68/141 | 6/17/84 |
| ***CD86*** | rs2681420 | AA/AG/GG | 42/102/53 | 26/53/25 |
|  | rs9836399 | GG/GA/AA | 14/84/116 | 8/38/61 |
|  | rs13064913 | GG/GA/AA | 4/44/164 | 2/26/79 |
|  | rs2001791 | CC/CT/TT | 38/108/67 | 21/53/33 |
| ***CD80*** | rs3915165 | AA/AC/CC | 17/79/117 | 7/47/53 |
|  | rs3915166 | CC/CT/TT | 17/79/118 | 7/47/53 |
|  | rs1852212 | AA/AG/GG | 17/79/117 | 7/47/53 |
|  | rs1880661 | CC/CT/TT | 11/75/126 | 4/40/63 |
|  | rs16829984 | CC/CG/GG | 23/81/110 | 11/46/50 |
|  | rs16829980 | GG/GA/AA | 18/77/118 | 7/47/53 |
|  | rs2228017 | TT/TC/CC | 19/79/115 | 6/40/61 |
|  | rs7628626 | AA/AC/CC | 0/36/177 | 1/17/89 |
|  | rs2692620 | CC/CT/TT | 18/79/116 | 6/40/61 |
|  | rs2629396 | CC/CA/AA | 34/94/84 | 16/45/46 |
|  | rs2049502 | CC/CT/TT | 24/106/81 | 18/47/42 |
|  | rs2222630 | GG/GA/AA | 18/77/119 | 7/47/53 |
|  | rs16829957 | GG/GA/AA | 43/107/63 | 20/52/35 |
|  | rs2670289 | CC/CA/AA | 23/78/111 | 6/42/59 |
|  | rs1485332 | GG/GC/CC | 27/99/88 | 17/41/49 |
|  | rs1599796^*^ | AA/AG/GG | 23/80/110 | 11/36/60 |
|  | rs17281703^*^ | AA/AG/GG | 0/46/166 | 0/19/88 |
| ***CD54*** | rs5498 | GG/GA/AA | 19/84/103 | 9/45/48 |
|  | rs5491 | TT/TA/AA | 2/25/186 | 1/14/92 |
| ***CD58*** | rs17036001 | AA/AG/GG | 1/20/193 | 0/13/94 |
| ***CD40L*** | rs3092923^*^ | CC/CT/TT | 8/29/177 | 2/5/100 |
| ***OX40L*** | rs1234315 | TT/TC/CC | 42/103/66 | 21/44/42 |
|  | rs10489266 | CC/CT/TT | 0/4/210 | 0/1/106 |
|  | rs1234314 | GG/GC/CC | 43/101/70 | 21/44/42 |
|  | rs10912564 | TT/TC/CC | 0/11/185 | 1/11/81 |
|  | rs17346501 | CC/CT/TT | 0/4/209 | 0/2/105 |
|  | rs10489267 | AA/AC/CC | 0/24/190 | 1/18/88 |
| ***CD40*** | rs752118 | TT/TC/CC | 18/84/98 | 4/52/47 |
|  | rs4239702 | TT/TC/CC | 18/105/89 | 15/52/40 |
|  | rs1535045^*^ | TT/TC/CC | 19/96/97 | 4/57/46 |
| ***OX40*** | rs3813201 | GG/GA/AA | 18/86/109 | 8/47/52 |
|  | rs2298209 | CC/CG/GG | 2/17/194 | 0/20/87 |
| ***CD2*** | rs798040^*^ | GG/GT/TT | 8/82/123 | 2/43/62 |
|  | rs699738^*^ | TT/TG/GG | 8/81/123 | 1/44/62 |
| ***CD28*** | rs3181094 | GG/GT/TT | 34/97/82 | 15/43/49 |
|  | rs1879877 | CC/CA/AA | 33/96/83 | 15/42/49 |
|  | rs3181096^*^ | TT/TC/CC | 12/59/142 | 8/29/70 |
|  | rs3181097 | AA/AG/GG | 54/102/58 | 32/46/29 |
|  | rs3181098^*^ | AA/AG/GG | 12/59/141 | 8/27/70 |
|  | rs3116492 | GG/GA/AA | 2/28/184 | 2/22/83 |
|  | rs3769686 | GG/GA/AA | 0/15/198 | 0/5/102 |
| ***CTLA4*** | rs11571315 | GG/GA/AA | 23/85/105 | 17/39/51 |
|  | rs4553808 | GG/GA/AA | 4/51/158 | 3/24/80 |
|  | rs11571316^*^ | TT/TC/CC | 7/57/149 | 9/25/73 |
| ***ITGB2*** | rs2070946 | GG/GA/AA | 1/40/171 | 1/29/77 |
|  | rs2838727 | TT/TC/CC | 1/54/158 | 0/19/88 |
|  | rs235331 | AA/AT/TT | 8/71/134 | 7/34/66 |
|  | rs2235133 | AA/AG/GG | 27/101/85 | 17/46/43 |
|  | rs2026882 | TT/TC/CC | 4/38/171 | 1/19/87 |
|  | rs3788150 | TT/TG/GG | 24/103/86 | 12/49/46 |
|  | rs2280965 | AA/AG/GG | 9/83/121 | 6/41/60 |
|  | rs170963 | AA/AG/GG | 10/71/132 | 5/41/61 |
|  | rs760462 | AA/AG/GG | 17/93/103 | 8/48/51 |
|  | rs2838735 | CC/CT/TT | 26/103/84 | 17/46/44 |
|  | rs684 | AA/AG/GG | 1/54/158 | 0/22/85 |

^a^ anti-HBs ≥ 1000 mIU/ml;

^b^ anti-HBs 10–99 mIU/ml.

^*^ deviated from Hardy-Weinberg equilibrium.
