# Supplementary material for: CD3Z Genetic Polymorphism in Immune Response to Hepatitis B Vaccination in Two Independent Chinese Populations
Source: PLoS One. 2012 Apr 18;7(4):e35303. doi: 10.1371/journal.pone.0035303 (PMC3329423; doi:10.1371/journal.pone.0035303)
Supplement: Table S4 — Confirmatory study in Shandong population. a HR: High-response group (anti-HBs ≥1000 mIU/ml). b LR: Low-response group (anti-HBs 10–99 mIU/ml). c P -values for Cochran-Armitage Trend test. (DOC) [file pone.0035303.s005.doc]

**Supplementary Table S4 confirmatory study in Shandong population**

| **Gene** | **SNP** | **Genotypes** | | | |  | **Alleles** | | | | |
| --- | --- | --- | --- | --- | --- | --- | --- | --- | --- | --- | --- |
|  |  |  | **HR ^a^**  n=1090 (100%) | **LR ^b^**  n=636 (100%) | ***P* ^c^** |  |  | **HR ^a^**  n=1090 (100%) | **LR ^b^**  n=636 (100%) | ***P*** | **OR (95%CI)** |
| *CD3Z* | rs12133337 | TT | 898 (82.4) | 498 (78.3) |  |  | T | 1976 (90.6) | 1124 (88.4) | **0.033** | 1.28 |
|  |  | CT | 180 (16.5) | 128 (20.1) | **0.035** |  | C | 204 (9.4) | 148 (11.6) |  | (1.01–1.61) |
|  |  | CC | 12 (1.1) | 10 (1.6) |  |  |  |  |  |  |  |
|  | rs10918706 | CC | 585 (53.7) | 319 (50.2) |  |  | C | 1596 (73.2) | 901 (70.8) | 0.132 | 1.13 |
|  |  | TC | 426 (39.1) | 263 (41.4) | 0.132 |  | T | 584 (26.8) | 371 (29.2) |  | (0.96–1.32) |
|  |  | TT | 79 (7.2) | 54 (8.5) |  |  |  |  |  |  |  |
| *OX40L* | rs10912564 | CC | 941 (86.3) | 557 (87.6) |  |  | C | 2024 (92.8) | 1189 (93.5) | 0.481 | 0.91 |
|  |  | TC | 142 (13.0) | 75 (11.8) | 0.487 |  | T | 156 (7.2) | 83 (6.5) |  | (0.68–1.20) |
|  |  | TT | 7 (0.6) | 4 (0.6) |  |  |  |  |  |  |  |

^a^ HR: High-response group (anti-HBs ≥ 1000 mIU/ml).

^b^ LR: Low-response group (anti-HBs 10–99 mIU/ml).

^c^ *P*-values for Cochran-Armitage Trend test.
