# Supplementary material for: CD3Z Genetic Polymorphism in Immune Response to Hepatitis B Vaccination in Two Independent Chinese Populations
Source: PLoS One. 2012 Apr 18;7(4):e35303. doi: 10.1371/journal.pone.0035303 (PMC3329423; doi:10.1371/journal.pone.0035303)
Supplement: Supplementary file 5 [file pone.0035303.s002.doc]

## Sheet: Sheet1

Supplementary Table S1 The details of candidate SNPs and the primer sequences for genotyping in first stage
Candidate gene	db SNP	Function	Forward primer	Reverse primer	Extend primer
CD4	rs11064391	5’near gene	ATTGTTGGAGTCGCAAGCTG	CAGAGTGAGAACCTGTCTTG	AAAGAAAAGAAAAGAAAAGAAAAC
	rs11064392	5’near gene	CAGAGCAAGGAGCTGTTTGT	AAAGAAGTGGGAGGCAGTTG	ggATGGGAACAGCAGTGG
	rs7956804	5’near gene	TTCTTTCCCCACAGCCTGGT	AGCGGCAAGAAAAAGATGGC	gggAAGAAAGATTCCATTAGTGGTAG
	rs2855534	5’near gene	TGCCATCTTTTTCTTGCCGC	AGCAGAATCAGGCTTAAATC	tcatGCTTAAATCAGGAGTCAAA
	rs1075837	Intron (Tag)	ATCTTGTCTCACCAGCAGGG	AGGATATAAAGTGGGCTCAG	aggtACTGCCTGGGGAAGG
	rs11064410	Intron (Tag)	GGAGGCAGAGTTATTTTAGG	TAATATCCTGCCATCACCCC	TCACCCCAAACGCAA
	rs2707212	Intron (Tag)	TCTGGGTTTGCAAGGCTCTG	AACCGTGCCCACCCTCCTT	tcCCCTCATCCCCTCAAA
	rs1075835	Intron (Tag)	ACATGCTCCTGGGTGTGTTC	AAGCTAGAGGAGGAGAGTTG	GAGTTGAGAAACCAAATCACTA
	rs3782736	Intron (Tag)	AGTGCATGTACGCTGATTG	CAAGGTGCAGAGAGGTTTAG	ggatAGGTTTAGTAACTCACCCAAG
	rs12812942	Intron (Tag)	CAATCTTTCCACCTCGATCT	AGGCCAGGCATGGTGGAAAT	GGCATGGTGGAAATCTCTCA
	rs10774451	Intron (Tag)	CTTTACAGGCATCATCTCAG	CAAAACAGTGTTGACAGTATC	ACAGTGTTGACAGTATCTATTTCTC
	rs1894290	Intron (Tag)	CAGAGGCCAAGAAACTAAGC	TTTTCCTCAAGCTGCCTAAG	CAGCATGTGCCTTTTG
	rs3213427	Intron (Tag)	CTGAACACTCCCCTGGGAT	TCCTTCAAGCCTAGCCCTTC	CCTAGCCCTTCTCTCAT
	rs3829972	Intron (Tag)	AGTACCTCCTGCCCCATGCT	GTTTGTGCAAGGTCAGGATG	GGATGTGAGTCTGTGG
	rs1045261	Intron (Tag)	TGGAAGCGAGCTTTGCTGG	CTGGCACAATCACCTTTGAG	agATCACCTTTGAGGTCCCTT
CD3G	rs2239695	5’near gene	GGCTTCGAGATGTGGCAGT	AAGGTGGCCTGAACTTTAGC	tCAACCACATTTACACACAA
	rs2071381	5’near gene	TCTGGCCCCTGAAAATGTGC	TTGAGATCATGAAAGAGGCG	GCAATAGGTGGAACAATTC
	rs7947185	5’near gene	CATCTGAGAGTCCCTCCTAA	GTTAGGATCGTAGCCAGTTG	actgGTTGCTAGTTGTTGTTGCT
	rs4544037	Intron (Tag)	GAAGTCAGGACAATGCTCAC	ATTACCAATGCCATTGAAG	agGCCATTGAAGCCCCTC
	rs1561966	Intron (Tag)	TGCACACTAGCAAGAAGCAC	ACCTGAGACCCTCAGCTTTC	ttcaTCCTACCATTATGTACCCAAT
CD3E	rs3825051	5’near gene	CTGTATTACTAGCACCCAGC	ACATTTTGATGAAATTCAC	ACTTATTTAATACATATTTTCTGTCTAC
	rs2231439	5’near gene	GCATTTGATTAATGCATGCT	GTGATAGTGTCAAAGTCTGC	GGTATGAGTGTGAATTGATG
	rs2231440	5’UTR	CATTTTCTGAAGCAGGCACC	CCAGAGGCCTCTCTACTTC	GCCTCTCTACTTCCTGTGTG
	rs1945764	Intron (Tag)	TGTGGGCAGTGTCTAATTCC	GGAAACAGCAGTAAGAAAAT	GGTTTAAGTTTAAATTGTAGGTAAAA
	rs3782042	Intron (Tag)	GTCAGGGCTAAATAATTCGC	TGAGCCAAATACTCAACACC	CTCAACACCAGCCAA
CD3D	rs2276424	Intron (Tag)	AAGAGTTCTAACCCTGCCTC	CTGTACTGAGCATCATCTCG	GGGGCTAAGAGAGGAGAAGAG
CD3Z	rs863454	5’near gene	GTACCAAGTCTGATGATGGG	ATGAAGAAAGTGAGTCCTGC	aTGAGTCCTGCAAATCA
	rs2995054	5’near gene	CAGACTTGGTACCTACTCAG	TTGCTGTGTGGCCTTGAGTA	gataTGTGGCCTTGAGTAAGTCAT
	rs2984800	5’near gene	CCCCAGCTGTGTCTTCATTT	GAAGGTTGTCATTGGTCTGG	ctTGCTGACACACTGATGAAGC
	rs1737502	Intron (Tag)	TGTGGACAGACCAGGTCATC	CTAAGCTGTTGCATTTGCAT	TTTGGAAATGCCCAGAT
	rs2995091	Intron (Tag)	GAGTATTGGAGGGAGAAGAG	CCCTCTGTCTCCATTTTACG	tcTTTCCTTTTTATTGGTTTCAC
	rs953809	Intron (Tag)	TAGGAGGTGGAGTTCTAGAC	CAGATAAATGATTCTGGGGC	agcgCCAGGTGAGCCCTTG
	rs1214606	Intron (Tag)	GCCAGAGAACTTTTCACAGG	CTCTTAGAAGGAGGCTCAAC	gggtAACTGTCTCAGCATGAATGA
	rs2280635	Intron (Tag)	CGGGAAGATGTTCACGTCTG	TATTGTCTCCCTGTGGGTAG	gggaaTCAGGCCAGGAGTACC
	rs858553	Intron (Tag)	TCAGTTAATTACCTGGTTG	CAAGAGGGCTGTGGATGTTG	tcccCTATGGAGCTGCCAAG
	rs2995088	Intron (Tag)	CTGCTGCTGTGTGCCTGAA	CCTTAGTTGCACTTTAGGTC	AGGTCAATGAATATCTAAATCACTC
	rs1773560	Intron (Tag)	AAAGACACAGGGAGAGATGG	TAGTAAGGATGGCCACCACA	gggtgTGACCACTCGTGAGG
	rs2995093	Intron (Tag)	ACCACAGGGATGCAATCAGC	TTGAACTGTACGAAGCTAGG	gaggAGCTAGGTTATTTGTGCCA
	rs10918706	Intron (Tag)	GGCCTGCTTTTTTCATCACC	AGGAGATCTCCATCAGAAGG	gggcAGGAGATAGCCCTGGAA
	rs704848	Intron (Tag)	TTCGATTCGTAAGCCACCTC	GTTCAAGAAGGAGGTTTAGG	tattTCCCCTACCTGGGGCTAAT
	rs12133337	Intron (Tag)	AGCTCCCTTGTGGGAAAAAC	TACCCTGCTTTCCCACCAAC	CCACCAACCTTGAAGA
	rs10918694	Intron (Tag)	TTCTCCCACCCCCTGGAAAA	ACAGGAGAAGGGGAGGATGA	cCTTCAGGCCTGCAGGGAGGGA
	rs12124119	Intron (Tag)	GTGAATTGGTCAGAACCAATC	ATTCTTCAGGCACACAGCAG	ACAGCAGCAGGAGGGAG
	rs2982484	Intron (Tag)	GATACCACCCAGGACTTTTG	GATTTCTCTCGACTCTGTGG	GGAATTTCTGAAGCTCTCT
	rs12036775	Intron (Tag)	TGGGAAATAGCCCACAGAAG	TTTGCTCGTATCCCAGTGAC	agcaTCCGTCTGGTCGGTTTAATAA
	rs3738212	3’UTR	GTCAGCCAGGGCCTGCGAG	TGGTACAGTTCAATGGTGCG	CAGAGGCCTGAGGCA
	rs1052231	3’UTR	ATCATCTTTGTGAAGGCCTC	TTTCCTGTCCTGCCACTGTC	GGCAGTTATAGGTCCCA
	rs870875	3’ near gene	ACTGCTGATTCTCAGGAGAG	CATTCCGGGCCGCAGGATG	ccgaCGCAGGATGCACCTGTGCTC
	rs870873	3’ near gene	CTCACACGCTGTCTATTCTG	CTTGTCAGGTGCAGGAAAAC	GGAAAACAAGCGCGA
CD86	rs2681420	Intron (Tag)	GAGTGTAGCAAAGACACAGG	CAATGTACAATTGCTTTTG	aAATGTACAATTGCTTTTGTTAGTA
	rs9836399	Intron (Tag)	TTTCTGCTTAGCACCTTGAG	CTAAGTCAGTGATCAGCTGG	GCTGGCACATCTACAGACCT
	rs13064913	Intron (Tag)	GAACTATTAGGCTCCATGAC	TCACCTGGAGGTGAATAGAC	tgagAGACAGTCGAGACCT
	rs2001791	Intron (Tag)	AGATGTGGACACTAAAGCCC	TGCCACTTAGTAGCTCTGTC	GATCTTGGGTAAATTACTTCA
CD80	rs16829988	5’near gene	TCCATCTCACCTCCAGTGTC	CCTACAATGTGTCTAGGAGC	GAGCATGACCTGTGG
	rs3915165	5’near gene	TGGCAGAGCTTAGTGGATAC	GCCTTCATGATTTCCAAAGC	gAAGCTTCAGTATTCCAAATCT
	rs3915166	5’near gene	GAAAGAGAGCAAACCGTGTG	CTGCCACAAACTTGTTGCTG	gggtgAGGAAGCCGCCGCTG
	rs1852212	5’near gene	AAAAAAAAAGACGCCATCC	AGTATTTCTCACCCACACGG	ccctaTCTCTTTCTGGCTGGT
	rs1880661	5’near gene	CTCTGAAGATGGTGGGATTC	ACTTTTGGGAGAGTGCTCAG	ggaaAGTGCTCAGCTCACA
	rs16829984	5’near gene	TGCTGTGTGATTTGCCCCAG	TGTTTGAAGAAGCGCCCTCT	tcGCTGCCGTCTTCTTCTA
	rs16829980	5’UTR	AGAGGCGACATTTCCCCTTC	AACTCCTGGCACTGACAAG	ggaagGGCACTGACAAGTACTGAG
	rs2228017	Exon	GCCAGCTCTTCAACAGAAAC	TGTTATCCACGTGACCAAGG	CCAAGGAAGTGAAAGAAGT
	rs7628626	Intron (Tag)	TATGGTGGGCACAGAAGTAG	AGTCCCTTCCAGAACTCAAG	aaaTCAAGAATTCTGCATTTCAAAAC
	rs2692620	Intron (Tag)	GAGATAATTTATGGCAAGCAC	GGATTCATATTTGGTGTGGG	gGGGTCTAGGCATTGAATT
	rs2629396	Intron (Tag)	TGCAGACATTCAAAGTATC	ATCAGGCACATACTTGTGGG	ACTTGTGGGCAACTAATA
	rs2049502	Intron (Tag)	TTTGAGCATCTCAGTTCACC	GAATATATACATCTTAGGC	GAATATATACATCTTAGGCCTATAA
	rs2222630	Intron (Tag)	CCTGGCCCTGGAGTACATA	GTCTATCCATAAAAAGGGAGG	ttACTGCAGACGTTACTTA
	rs16829957	Intron (Tag)	CCAGATCTCACATTCCTTAC	ATATAAGTGCCAACCATGGG	actCCAACCATGGGTTAAAC
	rs2670289	Intron (Tag)	GGACATGGCTCTTGCATTTG	TTCATTTGAGTGGCTATTGG	atTGGCTATTGGATTACTTTTCCTC
	rs1485332	Intron (Tag)	TTCCTGCACTCAAAGAGGTC	CTTTGCTTGTTCTACATGGG	aATGGGTTAGCTTTGCATTTCC
	rs1599796	3’UTR	CAGCAGAAAGGAGAACTATC	GACTACTGCTTTGACGTACC	gaTTTGACGTACCTAAATCATTC
	rs17281703	3’UTR	AACTGCCTTCCACAATTCAC	GATAATGTGATTATCCCAGC	TTATCCCAGCTTAAGTTGC
CD54	rs5498	Exon	TTGAGGGCACCTACCTCTGT	ACTCACAGAGCACATTCACG	CATTCACGGTCACCT
	rs5491	Exon	TGACATGCAGCACCTCCTGT	GGAGCAACTCCTTTTTAGGC	TCTCTATGCCCAACAAC
CD58	rs17036001	5’near gene	CTGGGAAGTGATATGACAGC	ATCCAAGCAATGCACTTCAC	ggggAATGCACTTCACACAGGG
CD40L	rs1800686	5’near gene	ATGGATGGGAAGTTGAGACG	CCCAACTCAGAATTTCGCTC	aTCTCTTTCAAAGGAAATTCCCT
	rs3092923	Intron (Tag)	TGTCAGAATGTGAACCATGC	TTCAGCCCACTGTAACACTG	aCCACTGTAACACTGTTACAAAGA
OX40L	rs1234315	5’near gene	GGCACAGATTTCTGATAGAC	CATTTGTTCAAATGCTAGAC	TGCTAGACTAGCCCA
	rs10489266	5’near gene	GTTCAAATGCTAGACTAGCC	CAGGCACAGATTTCTGATAG	GACTATCTAGGCATTCCA
	rs1234314	5’near gene	CCAGCATCCTTATGTTGTCC	ATGTAAAGTTCAAATGGGC	ggggTTTACTGAGTACATCACATGAG
	rs10912564	Intron (Tag)	TATTATCATTATCCATGAAC	TGTGTGCACACACATACTAT	gCAACATTTTAGTAGCAGTATGTTAA
	rs17346501	Intron (Tag)	CCTCTAGAACACAAGATCTC	CAGGCTACAACAATTCTGGG	aggaTACAACAATTCTGGGATTTAA
	rs10489267	Intron (Tag)	CTGGTTCACTAATCTAGAAAC	TCAGAATGAAAGGCCTACTG	AAAAAAGATTTTAGTTATGCCAG
	rs16845553	3’UTR	GGTACCTGACTACCATTTGC	ACTCCTTCACTCCTTGCTCC	cTTTTGATAATGCCACACACA
CD40	rs752118	5’near gene	ACGCATCTTGCTGTGTACGG	TTGGCCTTTGTGGGACCTG	ggggaAAGAGCTGTCTCTGGGA
	rs4239702	Intron (Tag)	TGTTTGCTTGTTGAAGGCCC	CAAAGGCTTGCTAATCCTTC	TTGCTAATCCTTCTTTTAAAACAAAAA
	rs1535045	Intron (Tag)	AATGGCTCTTAGGGAACAGG	TTCTCCACTCCTACCACAAG	CCTCTTTCCAGCTCC
OX40 	rs3813201	5’near gene	TCAGTGTCCAGCCTCCTGT	CTTAAGGTCACAGGTCCAAG	CAGGTCCAAGAAAGCCC
	rs2298209	3’UTR	GGTTTTTATTGTGGTCCCGC	TAGGTGCCGATGGCTGCCTC	cattGCTGCCTCCGGCTCT
CD2	rs798040	Intron (Tag)	TCTCTTGGAGATCACATGCC	GGAGGAAGGAACAGATTCAG	tcACAGATTCAGATGCACA
	rs699738	Exon	CCACAGAGTAGCTACTGAAG	GAAGTTGCTGGATTCTGAGG	agtGGTTGAAGCTGGAATTTG
CD28	rs3181094	5’near gene	GCAGTGGACCAGAATATTAG	GCATTAAAACAAGATTCATCC	cAAACAAGATTCATCCACATTCTTA
	rs1879877	5’near gene	ATTCATAACCACCACCCGAC	TGGCCGTGCTGGTGGAATAA	ggaTGGTGGAATAACCCTCTCT
	rs3181096	5’near gene	ATCTCAGAAATCCTGCCACG	GTCTCATGCTCTCTATGTCC	tttcCTATGTCCCTCAAGGTTAA
	rs3181097	5’near gene	CTATGTTCTTAGTGGGACAG	ACCTTGAGGGACATAGAGAG	ggggaAGACACCAAGGGGCTTTTG
	rs3181098	5’near gene	CCTTATGTCCTACACAAATG	GAGGAATTGGGAAACATCTG	GGGAAACATCTGCATAAA
	rs3116492	Intron (Tag)	GCACATGAAAAGATGCTTAG	TGGACGTGAAGTGATATCTC	tcagGGTTTGATTTGCTATTTCTCTAA
	rs3769686	Intron (Tag)	TATGCTGACCTAGGTCAAAG	CTGGCCATTGAAGGTTAAAG	ACTACTTTCTGCAGAGC
CTLA4	rs11571315	5’near gene	ACAGGCTGAACCACTGGCTT	CCCATGATCATGATAGAGTG	ggcGTGAGATCAATGCTGGA
	rs4553808	5’near gene	CTTCCTAATGCCAGACTGGG	CTGTGTGTTCCTCTTGAGGG	tcctGAACATTTGTTTTTCACTTTTT
	rs11571316	5’near gene	ATTGCTTGTTGGCGCTTGAG	CCTGCCTGTTTTCTATACAC	CACTGCTACACATTATAGAAA
ITGB2	rs2070946	5’near gene	TAGCACATTCTGCTACAGAG	TGGTTCCTCCGCTCCATTCT	tCTCCATTCTCTGGCTC
	rs2838727	Intron (Tag)	GATCACTCACACCGCAGGCAG	AAGAAAGCAAAGCCCTGAGC	tttgaAGCATTGTCCAGGCCTGCC
	rs235331	Intron (Tag)	GTACAAATATTAGAGTGGTG	TCAGCCTCCCGAGTATCTG	acctAGTATCTGGTACTACAGGCA
	rs2235133	Intron (Tag)	ATCATGCGCTGATTTCATTG	GCTTCATAAGGTTTGCCCAG	TGCCCAGGGTCACAAATTTAA
	rs2026882	Intron (Tag)	TCTACACCAGCACTGCCTTG	AGACATGCGCTGTGACTGAC	ACGTGTGCTGGAGAC
	rs3788150	Intron (Tag)	AGGGCACCAGAGGTGACAGT	GGATGGGAATGGGTAAGGCA	gGGGTAAGGCACAGATG
	rs2280965	Intron (Tag)	TCCCTGATTGGAATGTCACG	TCTCCCTCGGGTGCGGTGA	gtctGGTGCGGTGAGTTCTGT
	rs170963	Intron (Tag)	GATGATGTGTGGCCTCATGC	TGTGACTGACCCCCAGTAGA	GGCTCAAGAGCCTCC
	rs760462	Intron (Tag)	TCTCATTCCTTACCGTCAGC	TGGGCTGGAGGACCCAAAG	CCCAAAGCCGCTCCC
	rs2838735	Intron (Tag)	CTTCCTTTTTGCAACCCCGT	AAAGTTCCAATGGGTCTGCG	ttCCTCTGGTTCCTCAAGC
	rs684	3’UTR	CCAATTAACCAGAAATCCAG	GACGAGCCCCCAGAAGCAC	TGGCTGTCATTTTGAGGG

## Sheet: Sheet2

## Sheet: Sheet3
